# Goldenhar syndrome complicated with subglottic airway stenosis: a case report

**DOI:** 10.1186/s12871-023-02179-w

**Published:** 2023-06-16

**Authors:** Fei Xing, Xiao ming Deng, Dong Yang

**Affiliations:** Department of Anesthesiology, Plastic Surgery Hospital, Chinese Academy of Medical Sciences, Peking Union Medical College, 33 Ba-Da-Chu Road, Shi-Jing-Shan District, Beijing, 100144 People’s Republic of China

**Keywords:** Goldenhar syndrome, Subglottic airway stenosis, Chest computerized tomography

## Abstract

**Background:**

Goldenhar syndrome is a congenital disease that involves an absence or underdevelopment of structures that arise from the first and second pharyngeal arches and more or less severe extracranial anomalies. A variety of supraglottic malformations may be observed, including mandibular hypoplasia, mandibular asymmetry and micrognathia. Subglottic airway stenosis (SGS), which can cause difficulties in airway management during the perioperative period, is seldom emphasized in literature descriptions of Goldenhar syndrome, but can be clinically significant.

**Case presentation:**

An 18-year-old female with a history of Goldenhar syndrome presented for placement of a right mandibular distractor, right retroauricular dilator, and stage I transfer of a prefabricated expanded flap under general anesthesia. During tracheal intubation, the endotracheal tube (ETT) met resistance unexpectantly when attempting to pass through the glottis. Subsequently, we attempted the procedure with a smaller size ETT but again met resistance. With fiberoptic bronchoscope, we found that the whole segment of the trachea and bilateral bronchi were obvious narrow. Given the finding of unexpected severe airway stenosis and the associated risks with proceeding with the surgery, the operation was cancelled. We removed the ETT once the patient was fully awake.

**Conclusions:**

Anesthesiologists should be aware of this clinical finding when evaluating the airway of a patient with Goldenhar syndrome. Coronal and sagittal measurements on computerized tomography (CT) and three-dimensional image reconstruction can be used to evaluate the degree of subglottic airway stenosis and measure the diameter of the trachea.

## Background

Goldenhar syndrome is a congenital disease that results from defects in the development of the first and second branchial arches [1]. This defect occurs in 1:3500-1:5600 live births [1]. The etiology of Goldenhar syndrome is multifactorial and related to genetic and environmental factors. Some researchers suggest that the origin of this syndrome is due to the abnormal development of vascularization in 4th week of pregnancy when it comes to the development of the 1st and 2nd pharyngeal arches responsible for growth of craniofacial structures [1]. Autosomal dominant or recessive inheritance may contribute. Moreover, a lot of external factors like vasoactive medications, cocaine, thalidomide, or smoking can contribute to interference of normal growth of the first and second branchial arches [1, 2]. Craniofacial anomalies are typical clinical features of the syndrome, including mandibular hypoplasia, micrognathia, oral abnormalities, microtia, facial soft tissue and facial nerve underdevelopment [3]. A variety of extracranial anomalies may also be present, including central nervous system, skeletal, cardiac, lung, gastrointestinal, and kidney defects [4–6]. In Goldenhar syndrome, malformations of the respiratory system can include abnormal anatomy of the larynx and pharynx and lobular anatomy of the lungs [7–9]. The incidence of pulmonary agenesis is 1.4%.^10^ However, subglottic airway stenosis is seldom emphasized. Only 0.6% of patients were reported to have stridor [7, 10]. This clinical finding may cause difficulties in airway management during the perioperative period. We describe a case of a patient with Goldenhar syndrome with significant Subglottic stenosis (SGS). The Plastic Surgery Hospital ethics committee approved this case report. The patient provided written consent for the publication of this manuscript, which adheres to the applicable EQUATOR guideline.

### Case description

**Fig. 1:**
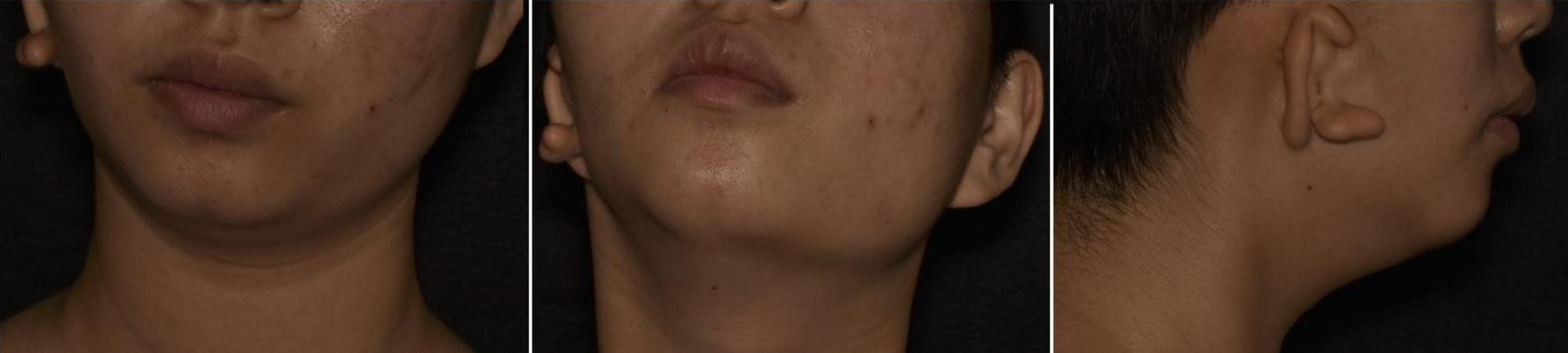
Physical examination of the patient showed right-sided facial asymmetry, microtia, a short mandible, and non-palpable temporomandibular joint

**Fig. 2:**
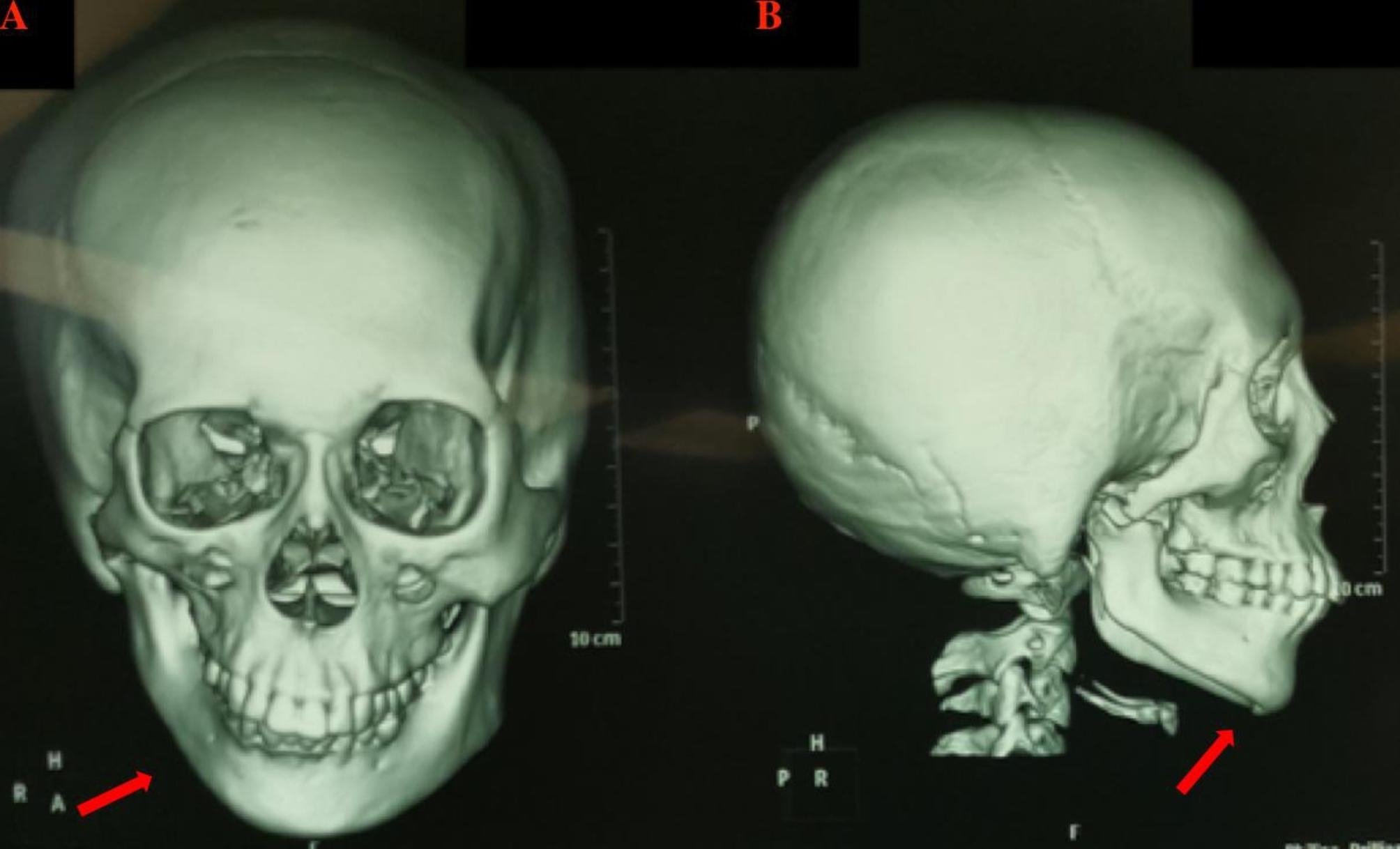
** A**: The cranial CT imaging of the patient shows that the right mandibular is shorter than the left. The arrow shows that right sided mandible hypoplasia. **B**: Right side of this patient

An 18-year-old female (weight, 53 kg, height, 162 cm, and BMI, 20.2 kg/m2) with a history of Goldenhar syndrome presented for placement of a right mandibular distractor, right retroauricular dilator, and stage I transfer of a prefabricated expanded flap under general anesthesia. The patient had no symptoms of respiratory disease, such as stridor, shortness of breathing, limitation on physical exertion, and no history of recurrent croup. No history of surgery and endotracheal intubation. On physical examination, the patient had right-sided facial asymmetry, microtia, a short mandible, and a nonpalpable temporomandibular joint (Fig. 1). Oral examination elucidated a Mallampati grade III score and 3.7 cm interincisal mouth opening. Cranial Computerized tomography (CT) imaging demonstrated right sided mandible hypoplasia (Fig. 2).

**Fig. 3:**
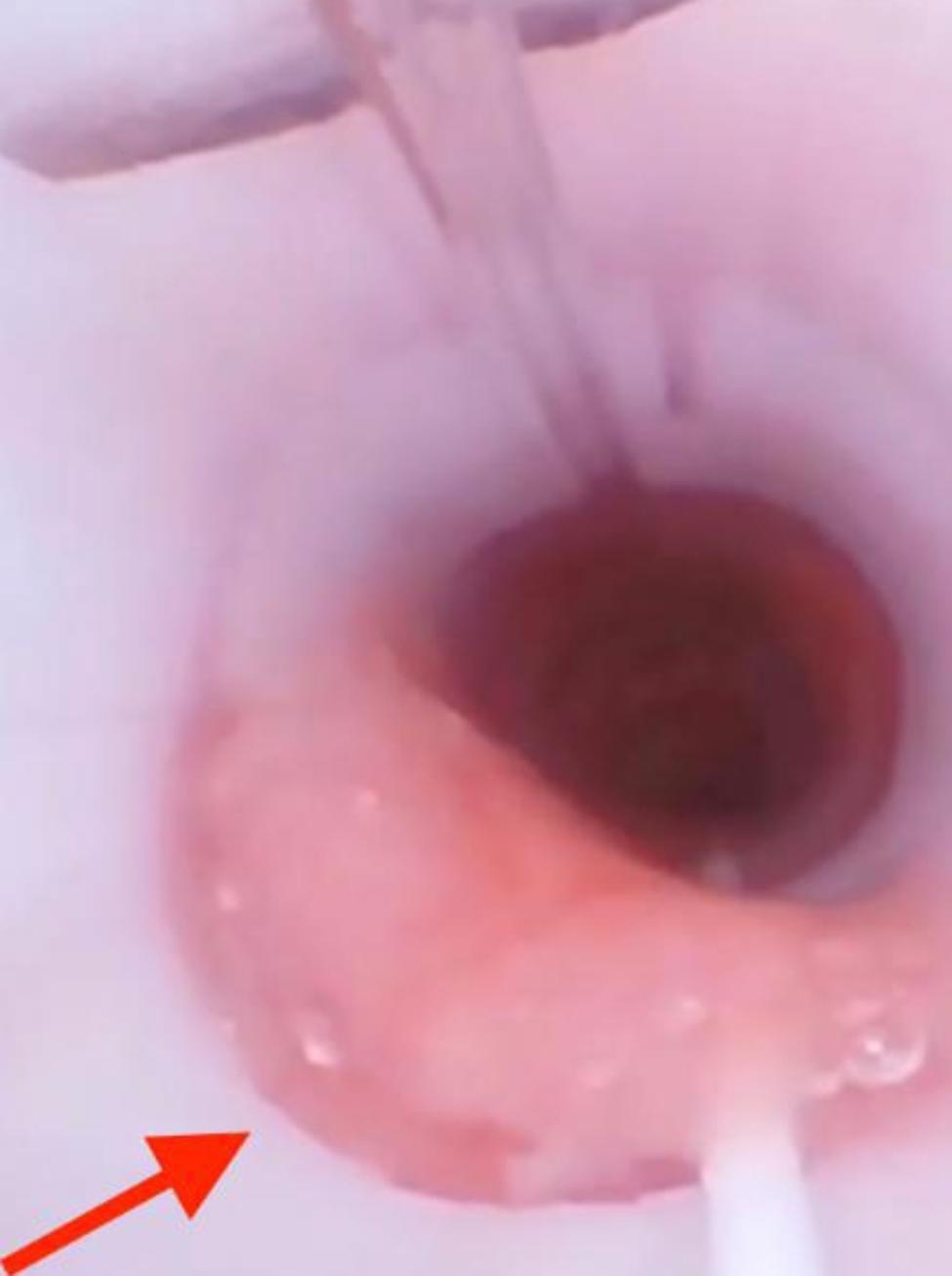
The tracheal mucosa protrudes from the lateral hole of the ETT. The outer diameter of 5-mm ETT is 6.8 mm

Standard ASA monitoring devices were applied and an intravenous induction of general anesthesia was proceeded. Given the oropharyngeal location of the operation and the patient’s asymmetric jaw deformity, a video laryngoscope was used to perform a transnasal endotracheal intubation with a 6.5-mm nasal right-angle cuffed endotracheal tube (ETT). A grade 1 Cormack-Lehane view of the glottis was obtained. The ETT smoothly passed through the nasal cavity, but met resistance unexpectantly when attempting to pass through the glottis. We then attempted the procedure with a 6.0-mm nasal right-angle cuffed ETT but again met resistance. Subsequently, the glottis and trachea were evaluated with a fiberoptic bronchoscope with confirmed evidence of subglottic airway stenosis. The patient was successfully intubated orally with a 5-mm cuffed endotracheal tube. However, there was no apparent ventilator leak despite tracheal balloon deflation. On further investigation with repeat fiberoptic bronchoscope evaluation, while the endotracheal tube was in the correct position, tracheal mucosa protruded from the lateral Murphy’s eye of the endotracheal tube (Fig. 3). The diameter of the patient’s airway was less than the outer diameter of 5-mm oral ETT with cuff (6.8 mm). Given the finding of unexpected severe airway stenosis and the associated risks with proceeding with the surgery, including airway injury or edema caused by repeated endotracheal intubation attempts, prolonged retention of the ETT after operation, and potential postintubation tracheal stenosis, the operation was cancelled. The ETT was secured while the patient was allowed to fully emerge from general anesthesia prior to extubation. After multiple attempts at intubation, the patient were promptly administered glucocorticoids to prevent and reduce airway edema. During the intubation attempts, the patient was well ventilated with a mask. We removed the ETT once the patient was fully awake. There was no evidence of any airway edema or respiratory compromise during the recovery in the post anesthesia care unit. Considering the potential risk of airway manipulation in general anesthesia, the procedure has been altered, which was allowed it to be performed under local anesthesia at a later date to avoid airway manipulation. Four days later, the patient underwent the placement of right retroauricular dilator, and stage I transfer of a prefabricated expanded flap under local anesthesia. The second portion will occur after further airway assessment and SGS management.

**Fig. 4:**
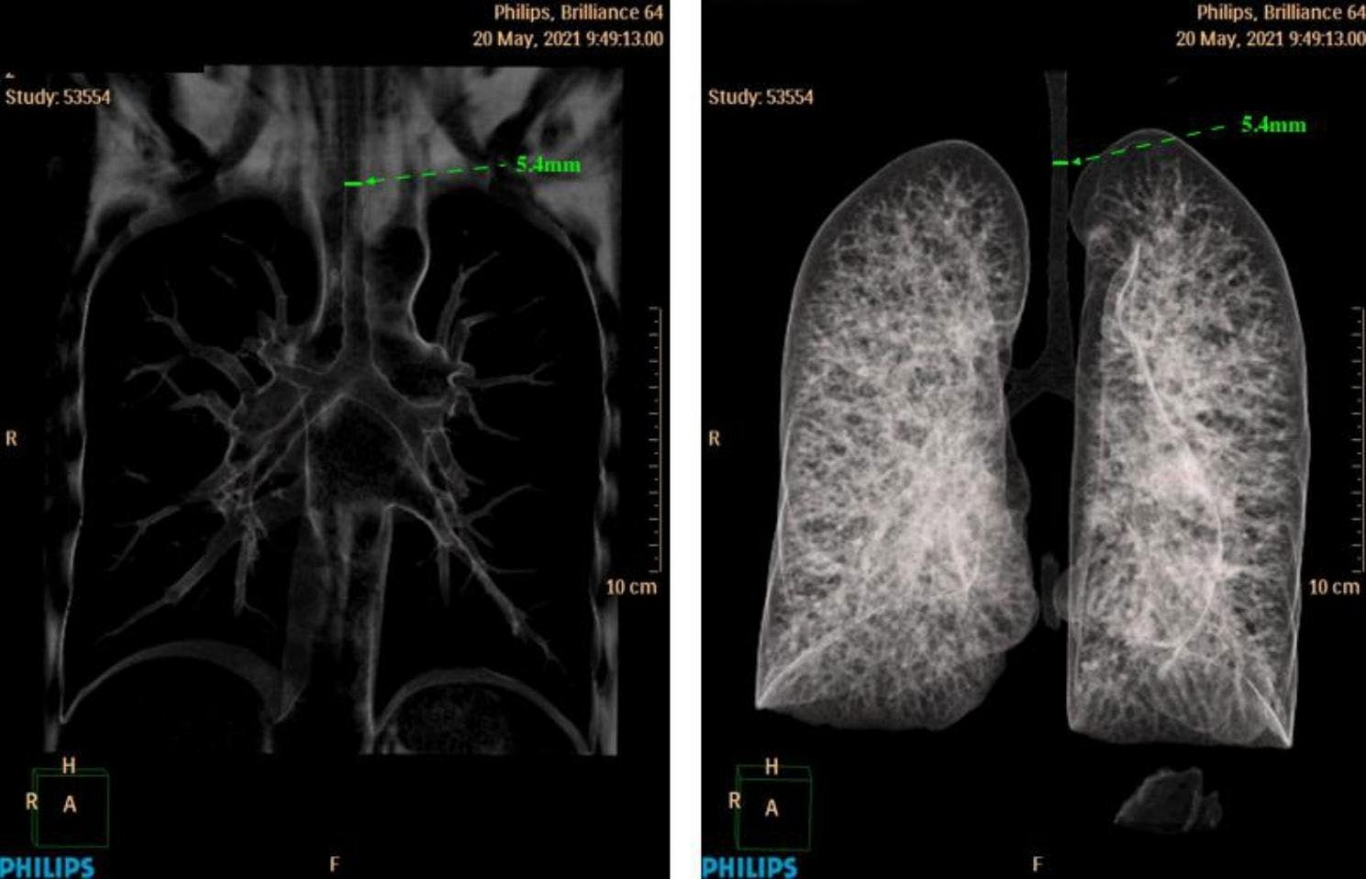
Three-dimensional image reconstruction of chest CT. The minimum diameter of the trachea was only 5.4 mm

**Fig. 5:**
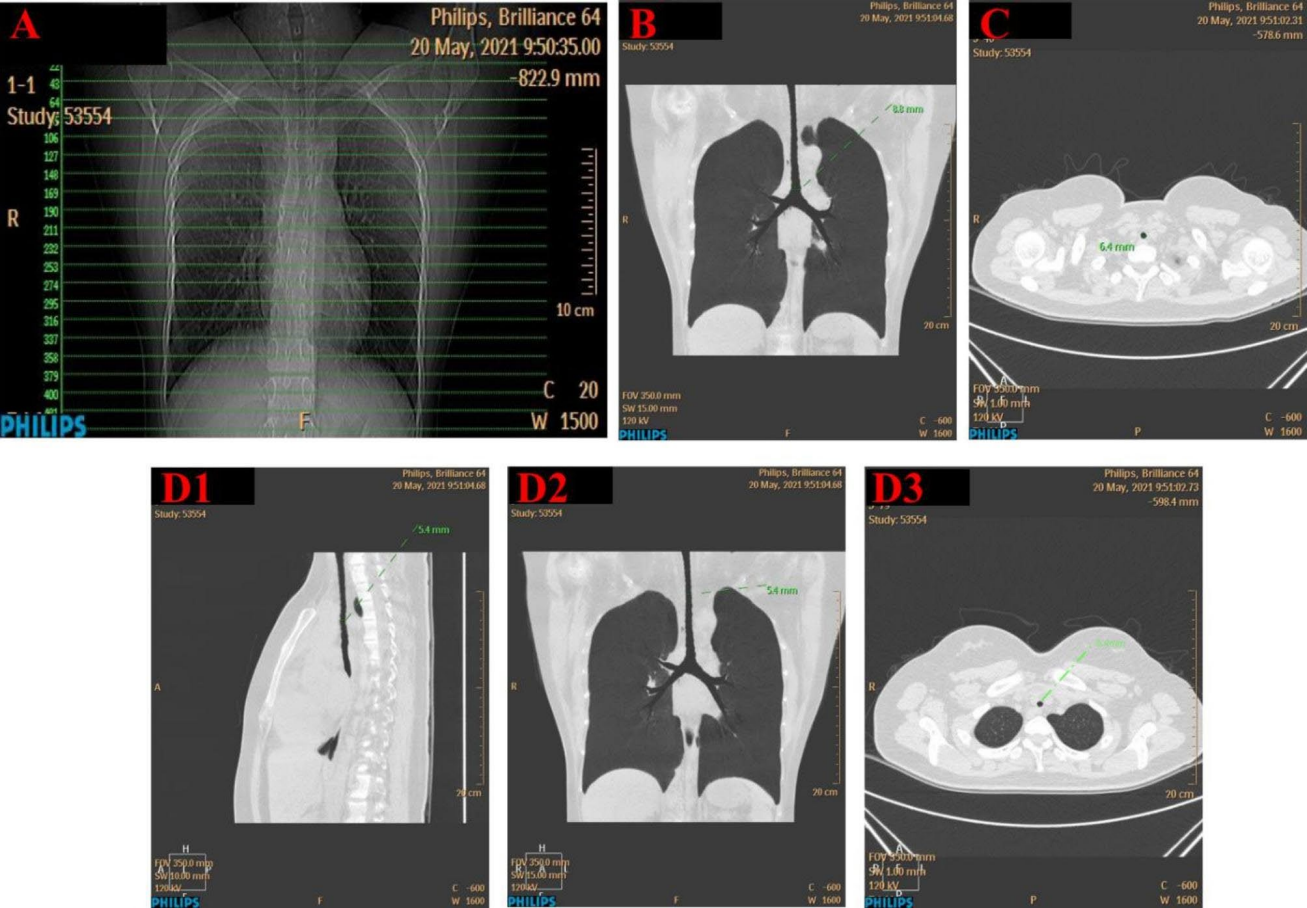
Coronal and sagittal measurements on CT and three-dimensional image reconstruction shows that the whole segment of the trachea and bilateral bronchi were all narrow. The minimum diameter of the trachea was only 5.4 mm. The maximum was 8.8 mm. The airway diameter in normal adults is approximately 15 mm ~ 20 mm

Chest CT provided detailed measurements of the subglottic airway. Through coronal and sagittal measurements on CT and three-dimensional image reconstruction, we found that the whole segment of the trachea and the bilateral bronchi were all narrow (Fig. 4). The minimum diameter of the trachea was only 5.4 mm, and the maximum was 8.8 mm; both were much narrower than normal (the airway diameter in normal adults is approximately 15 mm ~ 20 mm) [11] (Fig. 5). We recommend the patient go to the respiratory department for tracheoscopy, comprehensive evaluation of the degree and length of airway stenosis and assessment of the patient’s lung function after discharge. These results can be used as a reference for later surgeries to select anesthesia methods and tracheal tube models.

### Discussion and conclusions

Goldenhar syndrome involves an absence or underdevelopment of structures that arise from the first and second pharyngeal arches, and more or less severe abnormalities of extracranial anomalies [1]. The eformation in craniofacial area often cause upper airway obstruction and failure to thrive. Mandibular hypoplasia, mandibular asymmetry and micrognathia may constricting the oropharyngeal airway [1, 2]. Kourelis et al. and Jacobs et al. have observed that patients with GS will also have aberrant configuration of the nasopharynx involving pterygoid processes and adenoids, narrowing of the anteroposterior dimension of the airway at the level of the larynx or narrowing in the lateral dimension at the same level [1, 8]. Smith et al. described an infant with left pulmonary agenesis and complete tracheal rings for the length of the trachea [10]. Downing et al. reported on a premature infant with tracheoesophageal cleft, segmental tracheal stenosis with absence of cartilaginous rings, and left pulmonary agenesis [4]. However, the occurrence of subglottic stenosis in patients with GS have not been reported until now to our knowledge.

Airway stenosis is classified as congenital or acquired tracheal stenosis [11]. Acquired tracheal stenosis often involves SGS, which is usually caused by postintubation stenosis, burn injuries, and secondary healing after surgery [12]. Congenital tracheal stenosis is rare. According to the length of the affected area, congenital tracheal stenosis is classified into short section or long section stenosis. Tracheoscopy and CT could diagnose airway stenosis and can illustrate the extent of obstruction in the airway [12].

SGS is often difficult to identify before surgery because tracheoscopy and chest CT are not routine preoperative examinations without prior symptoms of airway stenosis. In addition, clinical findings including dyspnea and stridor may only be apparent when there is severe airway stenosis. We identified partial jaw and maxillofacial deformities in the preoperative examination and since the patient in this case did not show symptoms of airway stenosis, we had no concern initially for SGS.

Unexpected SGS may cause greater risks of anesthesia and surgery. If the size of the ETT is not suitable, a thicker ETT will compress the airway mucosa, resulting in ischemia and necrosis, and form a scar, which could increase the severity of SGS. To prevent long-term adverse outcomes, we suggest that patients with Goldenhar syndrome undergo chest CT before surgery to measure the airway diameter. If SGS is found, consider airway three-dimensional image reconstruction or tracheoscopy to evaluate the degree of stenosis, which can be classified by Myer-Cotton stenosis grades [11]. If the degree of SGS is serious, local anesthesia or monitored anesthesia care with local anesthesia and light sedation should be used to reduce airway manipulation and trauma. Delaying an elective procedure could be considered until the subglottic stenosis is managed. For patients with mild SGS utilize general anesthesia with suitably sized ETTs based on the narrowest diameter of the airway.

Multiple attempts at intubation might cause airway trauma. Foregoing additional attempts at intubation due to concern for significant subglottic stenosis and airway trauma until further work up can be done. A noninvasive method applied to maintain breathing and wait for the patient to wake up such as supraglottic airway or mask ventilation may be a better choice in this situation.

In conclusion, patients with Goldenhar syndrome not only have supraglottic malformations caused by craniofacial malformations, but may also have SGS. Therefore, during the preoperative evaluation for anesthesia, comprehensive physical and airway examination is necessary. We suggest that patients with Goldenhar syndrome undergo chest CT before surgery to measure the airway diameter. If SGS is found, consider airway three-dimensional image reconstruction or tracheoscopy to evaluate the degree of stenosis.

## Data Availability

Not applicable.

## Declarations

**Ethics approval and consent to participate**.

The Plastic Surgery Hospital ethics committee approved this case report.

**Consent for publication**.

Written informed consent was obtained from the patient for publication of this article and any accompanying images.

### Authors’ information (optional)

Authors and Affiliations.

Department of Anesthesiology, Plastic Surgery Hospital, Chinese Academy of Medical Sciences and Peking Union Medical College, Beijing, People’s Republic of China.

Fei Xing, Xiao ming Deng & Dong Yang.

## Abbreviations

SGS: Subglottic stenosis
ETT: Endotracheal tube
CT: Computerized tomography
